# Extracellular Vesicles From Liver Progenitor Cells Downregulates Fibroblast Metabolic Activity and Increase the Expression of Immune-Response Related Molecules

**DOI:** 10.3389/fcell.2020.613583

**Published:** 2021-01-12

**Authors:** Felix Royo, Mikel Azkargorta, Jose L. Lavin, Marc Clos-Garcia, Ana R. Cortazar, Monika Gonzalez-Lopez, Laura Barcena, Hernando A. del Portillo, María Yáñez-Mó, Antonio Marcilla, Francesc E. Borras, Hector Peinado, Isabel Guerrero, Mar Váles-Gómez, Unai Cereijo, Teresa Sardon, Ana M. Aransay, Felix Elortza, Juan M. Falcon-Perez

**Affiliations:** ^1^Center for Cooperative Research in Biosciences, Bizkaia Technology Park, Bizkaia, Spain; ^2^Centro de Investigación Biomédica en Red de Enfermedades Hepáticas y Digestivas, Madrid, Spain; ^3^Centro de Investigación Biomédica en Red de Cáncer, Instituto de Salud Carlos III, Madrid, Spain; ^4^ISGlobal, Hospital Clínic – Universitat de Barcelona, Barcelona, Spain; ^5^Health Sciences Research Institute Germans Trias i Pujol, Badalona, Spain; ^6^Institució Catalana de Recerca i Estudis Avançats, Barcelona, Spain; ^7^Departamento de Biología Molecular, Centro de Biología Molecular Severo Ochoa, Instituto de Investigaciones Sanitarias la Princesa, Universidad Autónoma de Madrid, Madrid, Spain; ^8^Àrea de Parasitologia, Departament de Farmàcia i Tecnologia Farmacèutica i Parasitologia, Universitat de València, Valencia, Spain; ^9^Joint Research Unit on Endocrinology, Nutrition and Clinical Dietetics, Health Research Institute La Fe, Universitat de València, Valencia, Spain; ^10^Department of Cell Biology, Physiology and Immunology, Autonomous University of Barcelona, Barcelona, Spain; ^11^REMAR-IVECAT Group-“Germans Trias i Pujol” Health Science Research Institute (IGTP), Badalona, Spain; ^12^Nephrology Department-“Germans Trias i Pujol” University Hospital, Can Ruti Campus, Badalona, Spain; ^13^Microenvironment and Metastasis Laboratory, Molecular Oncology Program, Spanish National Cancer Research Center, Madrid, Spain; ^14^Tissue and Organ Homeostasis, Centro de Biología Molecular “Severo Ochoa”, Universidad Autónoma de Madrid, Madrid, Spain; ^15^Spanish National Centre for Biotechnology, Spanish National Research Council, Madrid, Spain; ^16^Anaxomics Biotech, Barcelona, Spain; ^17^Ikerbasque, Basque Foundation for Science, Bilbao, Spain

**Keywords:** extracellular vesicles (EVs), exosomes, MLP29, fibroblast, cell crosstalk, immune response

## Abstract

Extracellular vesicles (EVs) mediate cell-to-cell crosstalk whose content can induce changes in acceptor cells and their microenvironment. MLP29 cells are mouse liver progenitor cells that release EVs loaded with signaling cues that could affect cell fate. In the current work, we incubated 3T3-L1 mouse fibroblasts with MLP29-derived EVs, and then analyzed changes by proteomics and transcriptomics. Results showed a general downregulation of protein and transcript expression related to proliferative and metabolic routes dependent on TGF-beta. We also observed an increase in the ERBB2 interacting protein (ERBIN) and Cxcl2, together with an induction of ribosome biogenesis and interferon-related response molecules, suggesting the activation of immune system signaling.

## Introduction

Extracellular vesicles (EVs) play an important role in cell-to-cell communication by interchanging messages between cells through their cargo and surface proteins (Fatima and Nawaz, 2017). Among their cargo, there are bioactive molecules capable of stimulating regenerative programs in damaged tissues (Fatima et al., 2017). In that regard, different types of EVs playing a role in liver regeneration and with anti-fibrotic effect have been described. Mesenchymal stem cell (MSC)-derived EVs mediate tissue regeneration in liver fibrosis (Katsuda and Ochiya, 2015; Damania et al., 2018). Moreover, they facilitate the recovery after ischemia in cardiac tissue, shortening the wound healing time, and reducing scar formation by modulating cellular migration, proliferation, and collagen synthesis (Zhao et al., 2015).

Liver stem cells constitute another population that secrete EVs with regenerative effects. Liver stem cell-derived EVs ameliorated renal function and morphology (Herrera Sanchez et al., 2014) and favor liver regeneration after hepatectomy in rats (Herrera et al., 2010) and after liver damage induced by non-alcoholic steatohepatitis (NASH; Bruno et al., 2020). They also show anti-tumoral effects (Brossa et al., 2020) and effectively reduce liver injury during hypoxic conditions created by normothermic machine liver perfusion associated to liver transplant, suggesting that liver stem cell-derived EVs can be employed to improve transplantation techniques (Rigo et al., 2018).

In the present work, we aimed to determine the effects of EVs released by MLP29, a cell line with characteristics of progenitor cell, isolated from fetal liver. During previous years, our group has characterized the proteome (Conde-Vancells et al., 2008), transcriptome (Royo et al., 2013), and metabolome (Royo et al., 2019b) of the EVs released by this cell line. Compared to EVs released by primary cultures of hepatocytes, MLP29-derived EVs are more resistant to mechanical stress (Royo et al., 2019b). Moreover, they can circulate in the organism to reach different organs, including liver, lung, spleen, and brain, according to studies of bio-distribution performed *in vivo* (Royo et al., 2019a). Given those characteristics, we hypothesize that MLP29-derived EVs are meant to have an effect over cellular parenchyma. To characterize the nature of such effect, we have chosen the 3T3-L1 fibroblasts, a cell line that can be differentiated into adipocytes and also has the ability to produce collagen and, therefore, mimic a fibrotic response (Li et al., 2019). We describe the crosstalk observed between these cell lines, as it could give information about the existing cell-to-cell communication in the organism by progenitor cells and fibroblasts.

## Materials and Methods

### Cell Lines

All media and reagents for tissue culture were purchased from GIBCO (Life Technologies Inc.). MLP29 is a murine liver progenitor cell line (Medico et al., 1996). The embryonic murine fibroblast cell line 3T3-L1 was purchased from ATCC (CL-173) as a murine embryonic fibroblast cell line.

### EVs Production and Purification

MLP29 cells were plated in 150-mm dishes, at 10 million cells per dish. Cells were cultured in complete Dulbecco’s modified Eagle medium medium [DMEM supplemented with 10% (v/v) fetal bovine serum (FBS), 0.1 mg/ml streptomycin, and 100 units/ml penicillin (GIBCO, Life Technologies Inc.)] for 24 h at 37°C and 5% of CO_2_. Then, cells were washed twice with Dulbecco’s modified phosphate-buffered saline (PBS) and incubated for 48 h (MLP29 cells) in 25 mM HEPES-containing complete DMEM medium [contaminating vesicles were first removed by overnight centrifugation at 110,000 × *g* (Thery et al., 2006)]. After incubation, media were collected, and EVs were isolated as previously described (Conde-Vancells et al., 2008). Briefly, culture supernatant was centrifuged at 1500 × *g* for 10 min to remove lifted cells and cellular debris. The resultant supernatant was centrifuged at 10,000 × *g* and 100,000 × *g* for 30 and 75 min, respectively. The resulting pellet was suspended in PBS, pooled, and again centrifuged at 100,000 × *g* for 75 min. The final pellet of EVs was suspended in PBS and stored at −80°C. Morphology, quantification, and the presence of typical EV markers are presented in Supplementary Figure 1. As control, EV preparations were obtained from similar amount of media that have never been in contact with cells, to ensure that the observed effect was not due to the media.

### EV Treatment of 3T3-L1

Approximately 2 million 3T3-L1 cells were treated with the EVs obtained from 200 million MLP29 cells. On average, this represents 20 μg of protein (measured by Bradford) contained in 8.9^∗^E10 particles (measured with Nanosight LM10) per treatment. A total of six plates were treated, with three different preparations of EVs and three preparations of controls, for 24 h, in 25 mM HEPES-containing complete DMEM medium (vesicles from tissue culture media were first removed by overnight centrifugation at 110,000 × *g*). At the end of the incubation, plates were washed twice with PBS, detached with Tryple (GIBCO), and then divided equally for proteomic and transcriptomic analyses. An aliquot was employed to measure cell viability, which was higher than 90% in all the cases. Trypsin was removed after pelleting the cells 1000 × x*g* for 5 min, which were frozen in dry ice and stored at −80°C until processing.

### RNA Library and Sequencing

Total RNA was extracted with RNeasy (Qiagen), including the step of DNase treatment for all the samples, and finally eluted in RNase-free water. The quantity and quality of the RNAs were evaluated using Qubit RNA HS Assay Kit (Life Technologies, Cat.# 32855) and Agilent RNA Nano Chips (Agilent Technologies, Cat.# 5067-1511), respectively.

Sequencing libraries were prepared using “TruSeq Stranded mRNA LT sample prep kit” (Illumina Inc., Cat.# RS-122-2101 or RS-122-2102), following the “TruSeq^®^ Stranded mRNA Sample Preparation LS Protocol (Part # 15031058 Rev. E). In brief, starting from 1000 ng of total RNA, mRNA was purified, fragmented, and primed for cDNA synthesis. cDNA first strand was synthesized with SuperScript-II Reverse Transcriptase (Life Technologies, Cat.#18064-014) for 10 min at 25°C, 15 min at 42°C, 15 min at 70°C, and paused at 4°C. cDNA second strand was synthesized with Illumina reagents at 16°C for 1 h. Then, A-tailing and adaptor ligation were performed. Finally, enrichment of libraries was achieved by PCR (30 s at 98°C; 15 cycles of 10 s at 98°C, 30 s at 60°C, 30 s at 72°C; 5 min at 72°C and paused at 4°C). Libraries were quantified and visualized on an Agilent 2100 Bioanalyzer using Agilent High Sensitivity DNA kit (Agilent Technologies, Cat.# G2938-90320) and Qubit dsDNA HS DNA Kit (Life Technologies, Cat.# 32851) and single-read sequenced to obtain 30 million 50-nt reads.

### Alignment and Quantification of Transcriptome

Quality Control of sequenced samples was performed by FASTQC software.^1^ Reads were mapped against the mouse (mm10) reference genome by STAR (Dobin et al., 2013) program to account for spliced junctions. The resulting BAM alignment files for the samples were then used to generate a table of raw counts by Rsubread (Liao et al., 2013). Raw counts table was the input for the differential expression (DE) analysis, carried out by DESeq2 (Love et al., 2014), to detect differentially expressed genes among the different conditions.

### Proteomic Analysis and Quantification

#### In Solution Digestion

Samples were digested following the filter-aided FASP protocol described by Wisniewski et al. (2009) with minor modifications. Trypsin was added to a trypsin:protein ratio of 1:10, and the mixture was incubated overnight at 37°C, dried out in a RVC2 25 speedvac concentrator (Christ), and resuspended in 0.1% formic acid (FA).

#### Liquid Chromatography

Liquid chromatography (LC) was performed using a NanoAcquity nano-HPLC (Waters), equipped with a Waters BEH C18 nano-column (200 mm × 75 μm ID, 1.8 μm). A chromatographic ramp of 120 min [5–60% acetonitrile (ACN)] was used with a flow rate of 300 nl/min. Mobile phase A was water containing 0.1% v/v formic acid, while mobile phase B was ACN containing 0.1% v/v FA; 0.5 μg of each sample were loaded for each run.

#### Mass Spectrometry

Peptides were eluted directly into the LTQ Orbitrap XL mass spectrometer through a nanoelectrospray capillary source (Proxeon Biosystems), at 300 nl/min and using a 120-min linear gradient of 3–40% ACN, followed up by an increase to 40% ACN for the next 30 min. The mass spectrometer automatically switched between MS and MS/MS acquisition in DDA mode. Full MS scan survey spectra (m/z 400–2,000) were acquired in the Orbitrap with mass resolution of 30,000 at m/z 400. After each survey scan, the six most intense ions above 1000 counts were sequentially subjected to collision-induced dissociation (CID) in the linear ion trap. Precursors with charge states of 2 and 3 were specifically selected for CID. Peptides were excluded from further analysis during 60 s using the dynamic exclusion feature.

#### Database Search

Database searching was performed using MASCOT 2.2.07 (Matrix Science, London, United Kingdom) against a UNIPROT/Swissprot database filled only with entries corresponding to *Homo sapiens* (without isoforms). For protein identification, the following parameters were adopted: carbamidomethylation of cysteines (C) as fixed modification, oxidation of methionines (M) as variable modifications, 10 ppm of peptide mass tolerance, 0.5 Da fragment mass tolerance, and up to two missed cleavage points, peptide charges of +2 and +3.

#### Progenesis LC-MS Software Analysis for Label Free Differential Expression Analysis

Progenesis LC-MS (version 2.0.5556.29015, Non-linear Dynamics) was used for the label-free differential protein expression analysis. One of the runs was used as the reference to which the precursor masses in all other samples were aligned to. Only features comprising charges of 2+ and 3+ were selected. The raw abundances of each feature were automatically normalized in a logarithmic scale against the reference run. Samples were grouped in accordance to the comparison being performed, and ANOVA analyses were performed. A peak list containing the information of all the features was generated and exported to the Mascot search engine (Matrix Science Ltd.). This file was searched against a UniProt/Swiss-Prot database under the conditions stated in the previous section, and the list of identified peptides was imported back to Progenesis LC-MS. Protein quantitation was performed based on the three most intense non-conflicting peptides (peptides occurring in only one protein), except for proteins with only two non-conflicting peptides. The significance of expression changes was tested at protein level, and proteins identified with at least two peptides and an ANOVA *p* value ≤ 0.05 were selected for further analyses.

### Annotation, Interaction, and Enrichment Pathway Analysis

The list of transcripts and proteins was annotated using the functional annotation [selecting GO Molecular Function (MF), Biological Process (BP) Cellular Component (CC), KEGG, and REACTOME] using DAVID 6.8^2^ (Huang da et al., 2009) and later assigning them manually to different groups of interest according to keywords shown in Tables 1, 2, and avoiding redundancy. Afterward, the list of gene/proteins was loaded into Cytoscape v.3.8.0^3^ (Shannon et al., 2003), and molecule interactions were retrieved from STRING App v.1.5.1 (Doncheva et al., 2019) with the confidence cutoff value of 0.4 and no addition of interacting molecules, against STRING experimental database, text-mining, and co-expression. Those nodes without edges were discharged, and the rest were clustered using the MCODE algorithms provided with clusterMaker2 v.1.3.1 (Morris et al., 2011). Finally, the clusters were manually annotated based on the enrichment groups obtained by functional enrichment analysis with STRING App v.1.5.1 (Doncheva et al., 2019) for each cluster (against GO Function, GO Components, GO Processes, Reactome pathways, and UniProt keywords) as shown in Figure 2. The functional pathway enrichment of Table 3 was performed using the Statistical Enrichment Test performed by Panther v.15.0^4^ (Mi et al., 2019). A Fisher exact test was used in order to determine whether the proportion of genes considered into the GO term or categories differed significantly between the dataset and the background [*p* adjusted (FDR method) < 0.05]. To determine the status of the pathway, fold change values were provided, and the category set employed was the GO Biological Processes.

**TABLE 1:**
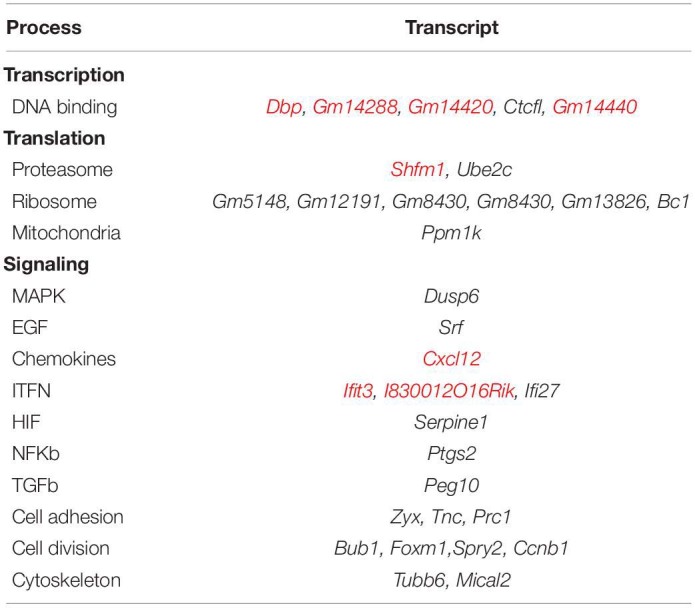
Transcripts regulated in 3T3-L1 cells after EV treatment, classified by their involvement in different cellular processes, according to DAVID 6.8 annotation of each transcript.

*In red, upregulated transcripts; in black, downregulated genes.*

**TABLE 2:**
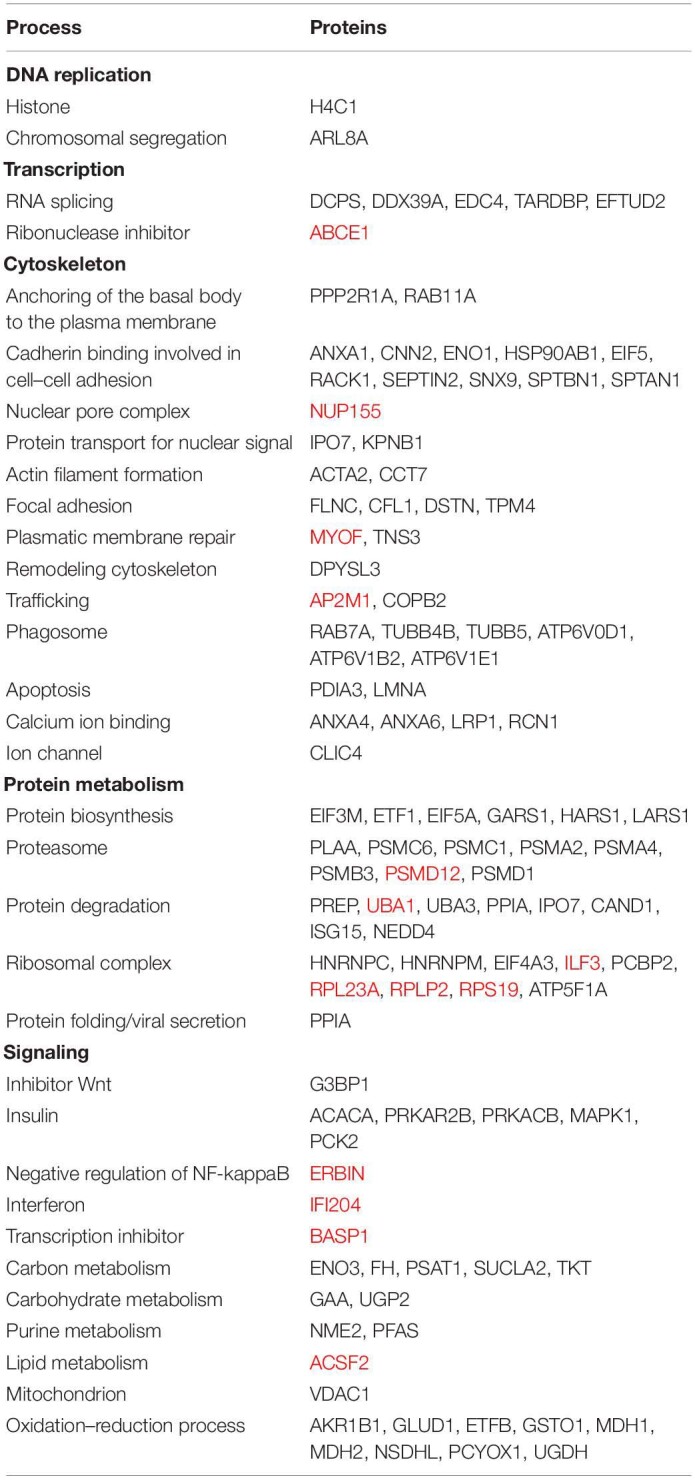
Proteins regulated in 3T3-L1 cells after EV treatment, classified according to their functional annotation in cellular processes, employing DAVID 6.8 database for the annotation of each protein.

*In red, upregulated proteins; in black, downregulated ones.*

**TABLE 3:**
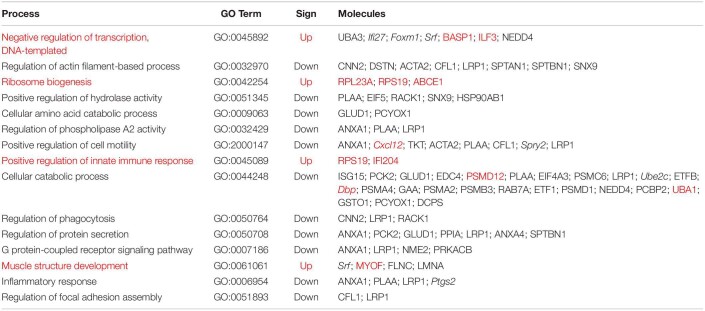
Most significant enriched pathways according to Panther v.15.0 Enrichment Analysis (GO Biological Processes).

*The software defines that each pathway was upregulated (red) or downregulated (black) according to the fold change values of each gene/protein group and their function. For the molecules’ annotation, red color indicates overexpression in fibroblast incubated with EVs, and black denotes downregulation with respect to untreated fibroblasts.*

## Results

### Transcriptomics

**FIGURE 1:**
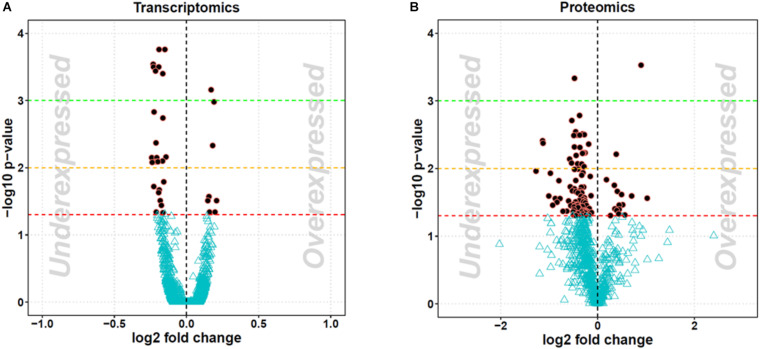
Volcano plot representing the changes in 3T3-L1 cells after MLP29-derived EV treatment. The graphs show fold changes (log2 transformed) versus *p* values (-log10 transformed). Significant regulated transcripts **(A)** and proteins **(B)** are represented as black dots in red circles. A gene or protein was considered regulated when its DE resulted in an adjusted *p* value < 0.05 (1.3 after −log10 transformation). Each experiment was performed in triplicate.

The treatment of 3T3-L1 cells with MLP29-derived EVs induced a change in the expression of 45 transcripts above the significance threshold (see Figure 1A). Most of those changes were downregulating the expression of genes involved in many active processes of the cell. In Table 1, we show the main cellular processes affected by EV treatment, while in Supplementary Table 1, we show the differential gene expression fold changes and *p* values. In addition, the data were deposited in the GEO repository under the access number GSE157022. A total of 45 transcripts were found significantly altered in EV-treated fibroblasts, and among them, 9 transcripts were upregulated. We found that *Cxcl12* and *Ifit3* were the most upregulated transcripts after treatment, while the predicted genes *Gm19592* and *Gm19820* were the most downregulated. Furthermore, the most downregulated protein coding gene was *Ptgs2*, a gene involved in inflammation response. Some of the transcripts were assayed by qPCR, and we confirm the upregulation of *Cxcl12* and *Ifit3*, as well as the downregulation of *Tubb6* and the same trend for *Peg10*, although in this case, the result was not significant (Supplementary Figure 3A).

### Proteomics

The treatment of 3T3-L1 cells with MLP29-derived EVs induced a change in the expression of different proteins above the threshold of significance (see Figure 1B). Most of those changes were a protein expression downregulation. In Table 2, we show the annotation of proteins modified, while in Supplementary Table 2, we show the differential protein expression with fold changes and *p* values. The expression of 107 proteins was affected by the EV treatment, and among them, 16 were upregulated, and the rest were downregulated after the treatment. The protein most over-represented was ERBIN, a protein that binds to the unphosphorylated form of the ERBB2 protein and regulates ERBB2 function and localization (Tao et al., 2014). The second most upregulated protein was ILF3, a protein with RNA binding capability involved in the formation of the ribosomal complex (Higuchi et al., 2012). Among the most downregulated proteins we found was RACK1, a scaffolding protein with participation in multiple functions such phagocytosis and cell-to-cell adhesion processes (Thorslund et al., 2011). The second most downregulated protein was MDH2, a mitochondrial malate dehydrogenase involved in ox–redox processes (Minarik et al., 2002). We should mention that regarding fold change value, the most downregulated protein was GLUD1, a mitochondrial glutamate dehydrogenase also involved in catabolism and TCA cycle (Tzimagiorgis et al., 1993;Coloff et al., 2016).

At a molecular level, we did not observe any overlapping between regulated genes and proteins. However, regarding the process affected, we encountered some similarities between the routes affected according to transcriptomic and proteomic data. In both cases, we observed downregulation for the machinery related to control of cytoskeleton and cell motility. We also observed downregulation of major signaling cascades (i.e., MAPK1, NFKb, TGFb, and EGF) in both types of analyses, while some interferon-related molecules were upregulated. We had performed a qPCR to see if the regulation of the observed proteins may reflect on the transcriptome, and we have observed the increase of *Myof* transcript, as well as the downregulation of *Plaa* and *Mdh1* transcripts, according to what was observed in the proteomics. However, this agreement was not clearly observed for Ccnb1 or Mapk1 (Supplementary Figure 3A). Interestingly, when 3T3 cells were treated with the vesicular fraction of the SEC-purified preparation of EVs (see Supplementary Materials and Methods), the downregulation of these last transcripts was observed (Supplementary Figure 3B), suggesting that some responses depend on the purity of the EV preparation, and to clearly visualize, one needs to remove protein aggregates that could unmask the response.

### Enrichment Analysis and Protein Networks

To get a better insight of the general effect achieved by regulations of genes and proteins, in this section, the corresponding analysis was performed considering both types of molecules as a single set. As described in the “Materials and Methods” section, the enrichment analysis performed with the Panther v.15.0 platform^5^ (Mi et al., 2019) takes into account both the gene/protein regulated, as well as the direction of such regulation, and accordingly, it calculates the probability for the pathways stored in the Gene Ontology Biological Processes database to be up- or downregulated. We performed this analysis with regulated genes/proteins, and the result is displayed in Table 3. The list includes a selection of pathways significantly regulated (*p*-adjusted <0.05), manually curated for redundancy and for lack of cellular context. The results indicate a reduction in genes related to transcriptional activity in fibroblasts treated with MLP29-derived EVs and upregulation of genes related to ribosome assembly, when compared to untreated cells. Interestingly, we also observed the upregulation in genes related to innate immunity. Some of the pathways that are downregulated included genes involved in migration, catabolism, inflammation, or phagocytosis.

**FIGURE 2:**
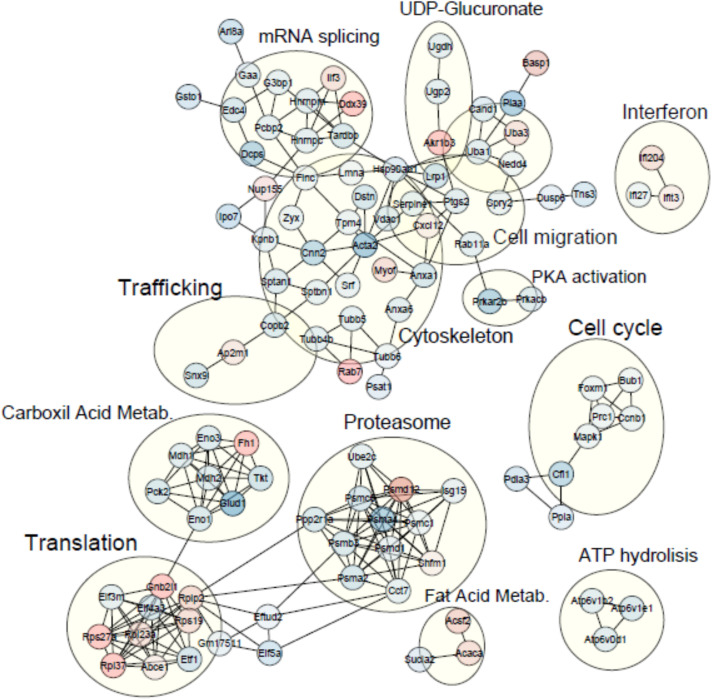
Clusters of protein interaction networks generated within the Cytoscape v.3.8.0 software environment. Red and blue color of the molecules denotes up- and downregulation, respectively. Annotation of the clusters was done manually according to functional enrichment generated with STRING App v.1.5.

To further explore the relationship between regulated proteins/genes, we loaded all regulated molecules in the Cytoscape v.3.8.0 software (Shannon et al., 2003). We retrieved protein interactions from STRING App v.1.5.1 (Doncheva et al., 2019) as described in the section “Materials and Methods,” and we discovered clusters of interacting molecules employing the MCODE algorithm from the clusterMaker2 App v.1.3.1 (Morris et al., 2011). Non-connected proteins were eliminated, and the annotations of the different clusters were decided according to the STRING functional enrichment annotations retrieved for each group of molecules, as well as protein individual annotation. Figure 2 illustrates the resulting interaction clusters, where the ribosome complex and the proteasome represent two well-defined groups. The machinery for mRNA splicing and different building blocks of the cytoskeleton appear as sub-groups within the largest cluster. Analysis in this interaction cluster showed that Cxcl2-Ptgs2 were related to regulation of immune activity and connected to other molecules related to cell migration. Other important groups were connected to cell cycling and the metabolism of carboxyl groups.

Regarding the functional meaning of the observed changes, the network presented in Figure 2 showed an increase in the presence of ribosome machinery, metabolism of fatty acids, and molecules related to interferon signaling. However, most of the cellular processes seemed to be inhibited, such as mRNA splicing, carbon metabolism, cell migration, the proteasome, and some molecules of the cytoskeleton. There was also a shortage in molecules involved in ATP hydrolysis. According to the results shown on the individual enrichment analysis, the interaction network suggested a slowdown of major cell activity, despite an increase of translation. We have observed as well a decrease in the metabolic activity measured by the MTT assay (Supplementary Materials and Methods and Supplementary Figure 2A) that could be explained by the downregulation described by the enrichment analysis.

## Discussion

In this work, we characterized the main effects produced by EVs derived from MLP29 liver progenitor cells in 3T3-L1 fibroblasts. The MLP29 cell line is an epithelial homogeneous cellular clone obtained by limiting dilution from a mouse embryonic liver cell line (Medico et al., 1996; Muller et al., 2002). This cell line responds to HGF with the whole array of its biological effects: scattering, survival, proliferation, and tubular morphogenesis (Medico et al., 1996). Moreover, their EVs carry a transcriptome enriched in signals regulating cell fate, proliferation, and cell organization (Royo et al., 2013). Accordingly, we analyzed the main changes in transcriptome and proteome in 3T3-L1 fibroblasts treated with MLP29-derived EVs. We should remark that purification of EVs through differential centrifugation could carry over other non-vesicular elements, which may also be responsible for the observed response, the reason why we have compared and further purified the preparation by SEC and observed a similar response to the fraction corresponding to vesicles. These results suggest that the observed effects are mainly due the vesicular component of our preparations (Supplementary Figures 2B, 3B).

We observed that treatment with MLP29-derived EVs promoted a decrease in pathways related to catabolic activity, cell proliferation, and cytoskeleton remodeling. It should be highlighted that the protein most differentially expressed in treated cells is ERBIN, an ERBB2 interaction protein blocking ERK activation (Huang et al., 2003). ERBIN is an intracellular protein of the LAP family (LRR and PDZ), containing an LRR domain and a PDZ domain (Borg et al., 2000; Kolch, 2003), and it is believed to play a role in basolateral targeting in epithelial cells (Dillon et al., 2002). The mechanism for inhibition of the route of TGF-β involves the formation of the ERBIN–MERLIN complex that disrupts the association between PAK2 and GTP-bound CDC42/RAC1. In fibroblasts, TGF-β activates p21-activated kinase 2 (PACK2), which was activated in response to TGF-β in different fibroblast cell lines, but not in epithelial cell lines (Wilkes et al., 2003; Wilkes et al., 2009). ERBIN also leads to deactivation of the ERK signal pathway, which is one of the main survival signaling pathways downstream HER2 (Yu et al., 2013).

We have also observed anti-apoptotic responses, such as the increase of the cytoskeleton protein myoferlin (MYOF), whose loss in fibroblasts is associated with a decrease in the expression of tight junction molecules and an increment in the number of cells positive for apoptotic markers (Leung et al., 2012). Taking into account that cells did not decrease their viability, the inhibitory responses observed do not lead to cell death. This agrees with previous studies of stem cell-derived EVs, showing the inhibition of fibrosis progression in models of kidney injury (Grange et al., 2019). Together, our results indicate that general cellular activity was slowed down in EV-treated compared to untreated fibroblasts, since transcription, cell motility, protein catabolism, and carbon metabolism were downregulated. Some enzymes with ATP hydrolytic activity were also downregulated. Consistently, we also detect reduction in metabolic activity measured by an MTT assay.

At the same time, there was an increase of signals related with immune system activation, centered in the activation of the transcription of the cytokine *Cxcl12*, a potent chemoattractant for hematopoietic cells. This molecule has been associated to fibrosis after radiation injury (Cao et al., 2019) and also to pro-metastatic stimulus of colon cancer cells through the PI3K/AKT/mTOR signaling pathway (Ma et al., 2017). However, in massive liver injury models where oval cell repair is involved, there is an upregulation of *Cxcl12* that interacts with oval cells because this cell type expresses CXCR4, the only known receptor for CXCL12 (Hatch et al., 2002).

The idea of a damage response is also reinforced by the observation of the increase of pathways related to ribosome biogenesis. These phenomena have been described to be associated to dsDNA sensing, a response to restrict virus reproduction and inflammatory response regulation (Bianco and Mohr, 2019). In this context, it is important to mention that MLP29-derived EVs contain dsDNA compatible with apoptotic features (Kruglik et al., 2019). Ribosome accumulation may also coordinate IFNB1 production, a pathway increased in fibroblasts that received MLP29-derived EVs. The upregulated molecules associated with the interferon pathway were *Ifit3* and IFI204 (Bianco and Mohr, 2019). To conform to the hypothesis of ribosome accumulation, we measure the changes of transcriptional activity of 45S pre-ribosomal RNA, observing a non-significant increase in cells treated by EVs by qPCR, and almost significant (*p* value 0.07) in cells treated with the vesicular fraction of our preparations (Supplementary Figure 3).

All the changes observed suggests that MLP29-derived EVs promoted somehow signals related to tissue damage in 3T3-L1 fibroblasts that may be partially explained by the presence of dsDNA (Bianco and Mohr, 2019). The effects inferred from changes in proteins and genes indicate a slowdown in some general cell activities including cell motility and cytoskeleton remodeling, transcription, and protein degradation machinery, carbon metabolism and TCA processes, and the upregulation of molecules related to innate immune response. Moreover, the results offer a new confirmation of the role of EVs in the crosstalk between liver stem and stromal cells in the liver.

## Data Availability Statement

The datasets generated for this study can be found the NCBI GEO acession GSE157022.

## Author Contributions

HAP, MY-M, AM, HP, IG, FB, MV-G, and JF-P originally conceived and designed the study. FR, MA, MG-L, and LB performed the experiments. JL and AM performed the transcriptomics analysis. MA and FE performed the proteomics analysis. FR, MA, JL, MC-G, AC, UC, and TS performed the enrichment analysis and data visualization. All authors participated in data interpretation, discussion, and manuscript drafting and editing.

## Conflict of Interest

The authors declare that the research was conducted in the absence of any commercial or financial relationships that could be construed as a potential conflict of interest.

## Footnotes

1 http://www.bioinformatics.babraham.ac.uk/projects/fastqc/

2 https://david.ncifcrf.gov/

3 https://cytoscape.org/

4 http://www.pantherdb.org/

5 http://www.pantherdb.org/

## Supplementary Material

Supplementary Figure 1 Characterization of EVs derived from MLP29.

Supplementary Figure 2 Measure of metabolic activity by MTT assay in 3T3.L1 cells after EV treatment.

Supplementary Figure 3 Changes in the transcription of 3T3.L1 cells induced by EV treatment measure by qPCR.

Supplementary Table 1 Significantly regulated transcripts.

Supplementary Table 2 Significantly regulated proteins.
