# Supplementary material for: Extracellular Vesicles From Liver Progenitor Cells Downregulates Fibroblast Metabolic Activity and Increase the Expression of Immune-Response Related Molecules
Source: Front Cell Dev Biol. 2021 Jan 12;8:613583. doi: 10.3389/fcell.2020.613583 (PMC7835421; doi:10.3389/fcell.2020.613583)
Supplement: Supplementary Figure 1 — Characterization of EVs derived from MLP29. [file Table_1.DOCX]

**Supplementary Material and Methods**

**EV characterization by electron microscopy and nanoparticle-tracking analysis**

For cryo-electron microscopy, EV preparations were directly adsorbed onto glow-discharged holey carbon grids (QUANTIFOIL, Germany). Grids were blotted at 95% humidity and rapidly plunged into liquid ethane with the aid of VITROBOT (Maastricht Instruments BV, The Netherlands). Vitrified samples were imaged at liquid nitrogen temperature using a JEM-2200FS/CR transmission electron microscope (JEOL, Japan) equipped with a field emission gun and operated at an acceleration voltage of 200 kV.

Size distribution within EV preparations was analyzed using the nanoparticle-tracking analysis (NTA), by measuring the rate of Brownian motion in a NanoSight LM10 system (Malvern, U.K.). The system was equipped with a fast video-capture and particle-tracking software. NTA post-acquisition settings were the same for all samples. Each video was analyzed to give the mean, mode, and median vesicle size, as well as an estimate of the concentration.

**Western blot analysis**

PBS-resuspended EVs were mixed with 10 µL of 4×NuPAGE LDS Sample Buffer (Thermo Fisher Scientific). The samples were incubated for 5 min at 37 ºC, 65 ºC, and 95 ºC, and separated on 4–12% pre-casted gels (from Thermo Scientific, Inc.). Antibodies were purchased from the following vendors: mouse monoclonal antibody against Aip1/Alix (49/AIP1) and GRP78 (40/BiP) were purchased from BD Biosciences; Armenian hamster anti-mouse CD81 (clone Eat2) was purchased from Bio-Rad. Mouse monoclonal antibodies against TSG101 (clone 4A10) and αTubulin (DM1A) were purchased from Abcam. Mouse monoclonal Anti-CD13 (3D8) was purchased from Santa Cruz Biotech., Inc. All the primary antibodies were diluted 1:1000.

**Measure of metabolic activity**

To measure cellular activity, the MTT assay was employed, a colorimetric assay where NAD(P)H-dependent cellular oxidoreductase enzymes reduce the tetrazolium dye MTT, 3-(4,5-dimethylthiazol-2-yl)-2,5-diphenyltetrazolium bromide to its insoluble formazan, which has a purple color. 3T3.L1 cells were plated in 96 wells in media depleted of vesicles, at a density of 10E5 cells per well, and incubated with 1ug of EVs per well for 24 h. Then, media was washed and replaced by new media with 0.5 mg/ml of MTT, incubated for 1 hour, and the washed, and cells lysed with 200 ul of a mix 1:1 of isopropanol and Dimethyl Sulfoxide. The absorbance was measured at 570 in an automated plate-reader spectrophotometer.

**Size-Exclusion Chromatography (SEC) purification of MLP29 derived EVs.**

SEC was performed by modifying an existing published protocol (1). Briefly, sepharose CL-2B (Sigma-Aldrich) was packed in a poly-prep chromatography column (BioRad) with 2 ml of bed volume. A preparation of MLP29 derived EVs of 20 ug in 200ul of PBS was allowed to enter in the column, and eluted with 2 ml of PBS, collecting fractions of 200 ul each. The number of vesicles and amount of proteins for each fraction was measured by NTA and Bradford techniques, respectively. Fractions 3 and 4 containing the vesicular fraction were pulled, while fractions 8 and 9 were pulled as non-vesicular but protein-containing fractions.

**Quantitative real time PCR analysis**

To extract RNA from cells, the RNAeasy mini kit (Qiagen) was employed. Twenty 20ul of cDNA was synthesized from 0.4–1 μg of RNA using qScript cDNA SuperMix (Quantabio) following the manufacturer's recommendations, and diluted for 1:5 in RNase & DNase free water. CDNA Primers sequences were obtained from the PrimerBank - MGH-PGA resource (<https://pga.mgh.harvard.edu/primerbank/>) with the following identification codes; Cxcl12 7305465a1; Ifit3 6754288a2, Tubb6 27754056a1, Peg10 31376257a1, Ccnb1 28195398a1, Myof 153791795c1, Mdh1 31982178a1, Mapk1 6754632a1, Plaa 114431249c1) while the primers for the 45S pre-ribosomal RNA were designed using primer blast function (https://www.ncbi.nlm.nih.gov/tools/primer-blast/) from the NCBI Reference Sequence: NR_046233.2. The Quantitative Real Time PCR was performed in Applied Biosystems QuantStudio 6 (Thermofisher) using the Applied Biosystems SYBR Green Master Mix (Thermofisher). Calculation of the expression was done employing the formula 2ΔCT, where for each gene, the Ct of each reaction is referred to the average of the Ct of control group.

**Reference**

(1)Böing AN, van der Pol E, Grootemaat AE, Coumans FA, Sturk A, Nieuwland R. Single-step isolation of extracellular vesicles by size-exclusion chromatography. J Extracell Vesicles. 2014 Sep 8;3. doi: 10.3402/jev.v3.23430. PMID: 25279113; PMCID: PMC4159761.

**Supplemental Figure 1**. **Characterization of EVs derived from MLP29**. The panel shows the presence of EVs markers by WB, the size profile characterized by NTA, and a typical image of MLP29 derived EVs by cryo-electron microscopy.


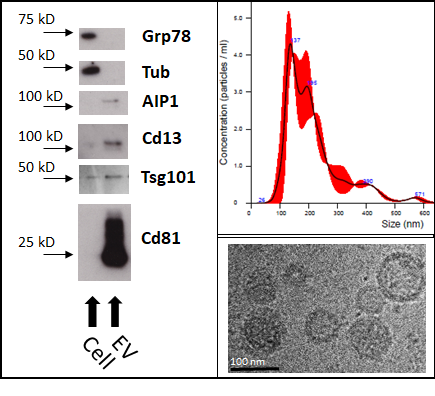


**Supplemental Figure 2. Measurement of metabolic activity by MTT assay in 3T3-L1 fibroblast after EV treatment**. Cells were treated in the same conditions described for transcriptomic and proteomics assays with EV preparation isolated by ultracentrifugation (A) or by the vesicular fraction (F3) or protein fraction (F8) of those preparation after further purification by SEC (B). Three independent replicates of EVs were assayed (A, B and C). Absorbance values were relativized to the average of the controls. The t-test calculated the p value corresponding to the difference between untreated cells and EV/vesicular fraction (F3) cells (n=3).


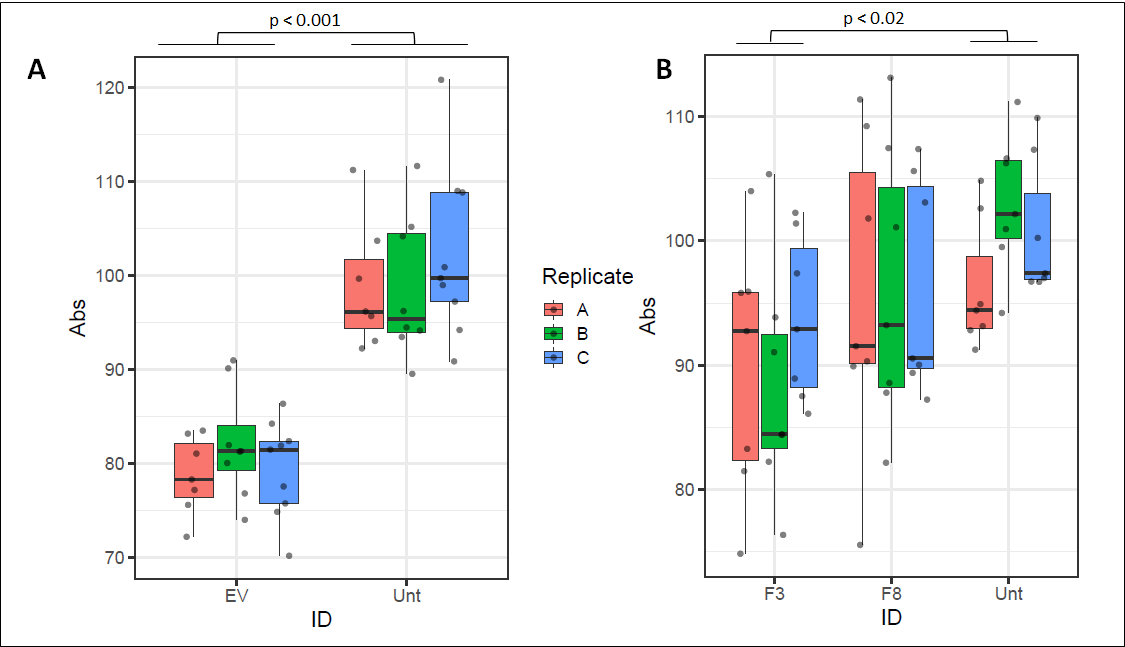


**Supplemental Figure 3.** **Changes in the transcription of 3T3-L1 cells induced by** **EV treatment** **measured by qPCR**. Cells were treated in the same conditions described for transcriptomic and proteomics assays with EV preparation isolated by ultracentrifugation (A) or by the vesicular fraction (F3) and protein fraction (F8) of those preparation after further purification by SEC (B). The statistic presented shows the p value corresponding to the difference between untreated cells and EV/vesicular fraction (F3) cells (n=3).


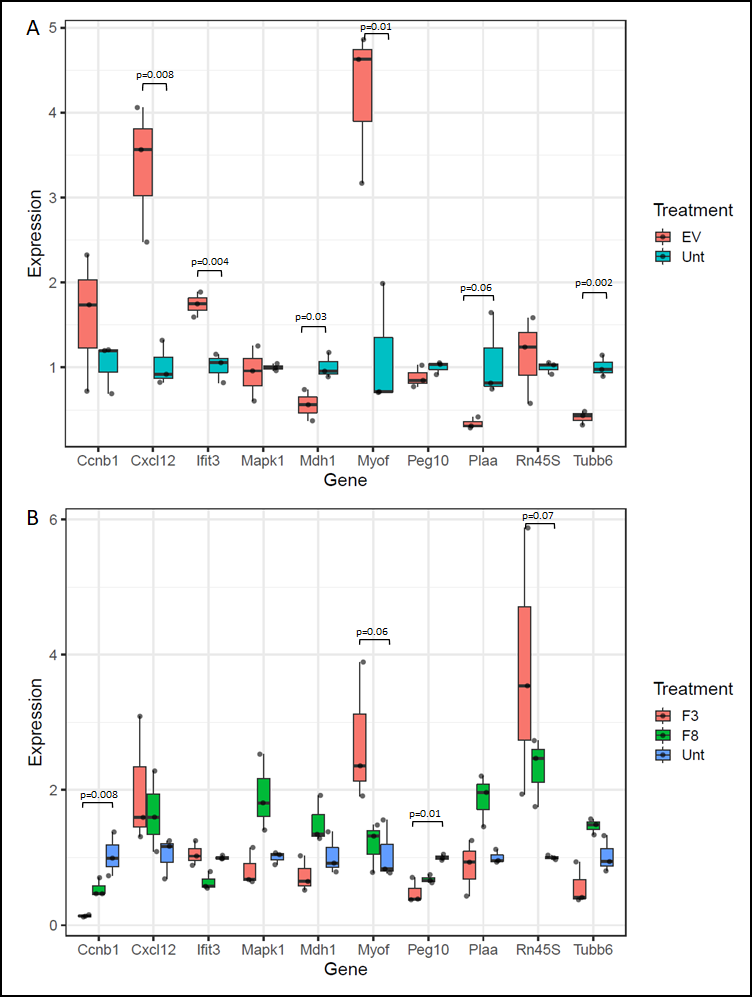


**Supplemental Table I**. Significative regulated transcripts, foldchange and adjusted p value.

| Gene_ID | hgnc_symbol | log2FC_EV *vs* NOEV | padj |
| --- | --- | --- | --- |
| 20315 | Cxcl12 | 0.17 | 0.0007 |
| 15959 | Ifit3 | 0.19 | 0.0011 |
| 667370 | I830012O16Rik | 0.18 | 0.0047 |
| 100503353 | Gm14440 | 0.16 | 0.0269 |
| 13999 | Gm14288 | 0.16 | 0.0269 |
| 628308 | Gm14420 | 0.15 | 0.0307 |
| 13170 | Dbp | 0.21 | 0.0307 |
| 626877 | Gm14405 | 0.20 | 0.0455 |
| 20422 | Shfm1 | 0.16 | 0.0459 |
| 100503203 | Gm19592 | -0.59 | 3.55E-25 |
| 100503665 | Gm19820 | -0.42 | 3.43E-11 |
| 100503279 | NA | -0.37 | 3.43E-11 |
| 19225 | Ptgs2 | -0.33 | 4.72E-06 |
| 100861862 | NA | -0.28 | 1.63E-05 |
| 18787 | Serpine1 | -0.27 | 1.63E-05 |
| 68612 | Ube2c | -0.27 | 2.76E-05 |
| 100503344 | Gm19645 | -0.24 | 1.32E-05 |
| 100568459 | Bc1 | -0.24 | 0.0071 |
| 664799 | Ctcfl | -0.24 | 0.0083 |
| 21923 | Tnc | -0.24 | 0.0000 |
| 667035 | Gm8430 | -0.23 | 0.0003 |
| 20807 | Srf | -0.23 | 0.0003 |
| 67603 | Dusp6 | -0.23 | 0.0189 |
| 170676 | Peg10 | -0.22 | 0.0015 |
| 100861897 | NA | -0.22 | 0.0000 |
| 100502641 | Gm17511 | -0.22 | 0.0082 |
| 22793 | Zyx | -0.21 | 0.0004 |
| 268697 | Ccnb1 | -0.21 | 0.0043 |
| 24064 | Spry2 | -0.21 | 0.0455 |
| 243382 | Ppm1k | -0.21 | 0.0071 |
| 100503298 | Gm19631 | -0.20 | 0.0000 |
| 666899 | Gm12191 | -0.20 | 0.0082 |
| 100502827 | Gm19400 | -0.19 | 0.0003 |
| 100503054 | Gm19528 | -0.19 | 0.0002 |
| 233406 | Prc1 | -0.19 | 0.0213 |
| 100502825 | Gm13826 | -0.18 | 0.0232 |
| 320878 | Mical2 | -0.18 | 0.0307 |
| 52668 | Ifi27 | -0.17 | 0.0162 |
| 14235 | Foxm1 | -0.17 | 0.0360 |
| 67951 | Tubb6 | -0.17 | 0.0079 |
| 100504337 | Gm20181 | -0.16 | 0.0004 |
| 100861649 | NA | -0.16 | 0.0018 |
| 12235 | Bub1 | -0.16 | 0.0473 |
| 100861991 | NA | -0.15 | 0.0002 |
| 381438 | Gm5148 | -0.14 | 0.0069 |

**Supplemental Table 2.** Significative regulated proteins,

| UNIPROT ID | log2FC_EV *vs* NOEV | pvalue |
| --- | --- | --- |
| Erbin | 0.90 | 0.0003 |
| Ilf3 | 0.38 | 0.0061 |
| Abce1 | 0.18 | 0.0146 |
| Uba3 | 0.35 | 0.0177 |
| Ifi204 | 0.41 | 0.0218 |
| Rplp2 | 0.49 | 0.0242 |
| Basp1 | 0.70 | 0.0254 |
| Psmd12 | 1.02 | 0.0275 |
| H4c1 | 0.52 | 0.0344 |
| Rps19 | 0.44 | 0.0347 |
| Nup155 | 0.36 | 0.0394 |
| Acaca | 0.44 | 0.0403 |
| Rpl23a | 0.39 | 0.0417 |
| Myof | 0.43 | 0.0468 |
| Acsf2 | 0.56 | 0.0486 |
| Ap2m1 | 0.27 | 0.0496 |
| Rack1 | -0.48 | 0.0005 |
| Mdh2 | -0.37 | 0.0016 |
| Api5 | -0.53 | 0.0020 |
| Tns3 | -0.45 | 0.0029 |
| Ppia | -0.31 | 0.0031 |
| Eif3m | -0.27 | 0.0032 |
| Eno1 | -0.42 | 0.0032 |
| Hsp90ab1 | -0.37 | 0.0032 |
| Anxa1 | -0.49 | 0.0033 |
| Pcyox1 | -1.14 | 0.0039 |
| Acta2 | -1.13 | 0.0042 |
| Mapk1 | -0.19 | 0.0044 |
| Gsto1 | -0.48 | 0.0048 |
| Vdac1 | -0.37 | 0.0049 |
| G3bp1 | -0.26 | 0.0059 |
| Pcbp2 | -0.29 | 0.0059 |
| Rab11a | -0.31 | 0.0060 |
| Ppp2r1a | -0.44 | 0.0064 |
| Arl8a | -0.58 | 0.0072 |
| Tkt | -0.54 | 0.0083 |
| Etfb | -0.41 | 0.0085 |
| Psat1 | -0.33 | 0.0086 |
| Tubb4b | -0.28 | 0.0094 |
| Tubb5 | -0.28 | 0.0094 |
| Ddx39a | -0.40 | 0.0102 |
| Psmc6 | -0.48 | 0.0103 |
| Glud1 | -1.27 | 0.0109 |
| Hnrnpm | -0.31 | 0.0114 |
| Prep | -0.30 | 0.0115 |
| Psma4 | -0.97 | 0.0117 |
| Dpysl3 | -0.33 | 0.0125 |
| Rab7a | -0.15 | 0.0131 |
| Pck2 | -0.45 | 0.0150 |
| Cfl1 | -0.80 | 0.0151 |
| Lrp1 | -0.56 | 0.0186 |
| Hars1 | -0.29 | 0.0188 |
| Atp6v0d1 | -0.34 | 0.0189 |
| Pdia3 | -0.48 | 0.0199 |
| Akr1b1 | -0.36 | 0.0212 |
| Etf1 | -0.52 | 0.0214 |
| Psmb3 | -0.45 | 0.0219 |
| Cand1 | -0.36 | 0.0224 |
| Uba1 | -0.33 | 0.0228 |
| Sptan1 | -0.41 | 0.0230 |
| Flnc | -0.14 | 0.0252 |
| Plaa | -1.01 | 0.0255 |
| Sucla2 | -0.34 | 0.0258 |
| Cct7 | -0.27 | 0.0270 |
| Dcps | -0.87 | 0.0277 |
| Cnn2 | -0.78 | 0.0278 |
| Anxa6 | -0.30 | 0.0284 |
| Prkacb | -0.38 | 0.0297 |
| Kpnb1 | -0.26 | 0.0298 |
| Ipo7 | -0.60 | 0.0301 |
| Gaa | -0.29 | 0.0310 |
| Ugdh | -0.29 | 0.0310 |
| Edc4 | -0.46 | 0.0311 |
| Clic4 | -0.84 | 0.0315 |
| Isg15 | -0.25 | 0.0321 |
| Eif5a | -0.50 | 0.0339 |
| Nsdhl | -0.41 | 0.0340 |
| Prkar2b | -0.93 | 0.0348 |
| Eif4a3 | -0.44 | 0.0349 |
| Psmd1 | -0.23 | 0.0350 |
| Gars1 | -0.55 | 0.0353 |
| Atp6v1e1 | -0.42 | 0.0353 |
| Septin2 | -0.31 | 0.0363 |
| Dstn | -0.49 | 0.0369 |
| Eno3 | -0.39 | 0.0375 |
| Copb2 | -0.40 | 0.0377 |
| Nedd4 | -0.17 | 0.0390 |
| Snx9 | -0.49 | 0.0406 |
| Ugp2 | -0.31 | 0.0415 |
| Nme2 | -0.64 | 0.0427 |
| Fh | -0.23 | 0.0427 |
| Pfas | -0.25 | 0.0428 |
| Eif5 | -0.71 | 0.0428 |
| Hnrnpc | -0.29 | 0.0431 |
| Anxa4 | -0.50 | 0.0435 |
| Iars1 | -0.33 | 0.0436 |
| Tpm4 | -0.21 | 0.0442 |
| Psmc1 | -0.17 | 0.0446 |
| Lmna | -0.13 | 0.0447 |
| Rcn1 | -0.26 | 0.0451 |
| Atp5f1a | -0.40 | 0.0464 |
| Atp6v1b2 | -0.31 | 0.0467 |
| Psma2 | -0.50 | 0.0482 |
| Eftud2 | -0.18 | 0.0486 |
| Sptbn1 | -0.24 | 0.0488 |
| Tardbp | -0.45 | 0.0497 |
| Mdh1 | -0.34 | 0.0499 |
